# Impact of migration on diabetes burden: audit in the metropolitan area of Bologna, Italy

**DOI:** 10.1007/s40618-023-02157-6

**Published:** 2023-07-20

**Authors:** G. Marchesini, D. Gibertoni, C. Giansante, V. Perlangeli, R. Grilli, L. Scudeller, C. Descovich, P. Pandolfi

**Affiliations:** 1 IRCCS Azienda Ospedaliero-Universitaria di Bologna, Bologna, Italy; 2 Department of Public Health, Local Health Authority of Bologna, Bologna, Italy; 3 Evaluation and Policy Unit, U.O. Health Services Research, Local Health Authority of Romagna, Ravenna, Italy

**Keywords:** Diabetes risk, Drug treatment, Incident diabetes, Metabolic control, Migrants, Protocol adherence

## Abstract

**Purpose:**

To investigate the impact of diabetes in immigrants on the Italian healthcare system, as well as their compliance with standard protocols of control and treatment.

**Methods:**

The prevalence of immigrants with diabetes living in the metropolitan area of Bologna (about 1 million inhabitants) in 2019 was investigated using a database containing all subjects in active follow-up for diabetes, based on antidiabetic drug use, disease-specific copayment exemption, ICD-9 codes, continuous care in diabetes units. Country of origin was derived from fiscal code.

**Results:**

The overall prevalence of diabetes (n = 53,941; 51.8% males, median age 64) was 6.1% in both Italy-born and immigrant cohorts. Immigrant prevalence was 12.4%, moderately higher than that observed in the total population (12.2%). Diabetes risk was increased in the whole immigrant cohort (odds ratio (OR) 1.74; 95% Confidence Interval (CI) 1.69–1.79). Among cases with incident diabetes, the proportion of immigrants (median age, 49 vs. 65 in Italy-born individuals) increased progressively from 11.7% to 26.5% from 2011 to 2019 (males, 8.9–21.0%; females, 14.9–32.8%) in all age groups, particularly in young adults, but also in older subjects. Metabolic control was lower in immigrants, as was adherence to shared diagnostic and therapeutic protocols, without systematic differences in antidiabetic drug use, but much lower use of drugs for comorbid conditions.

**Conclusions:**

The population with diabetes in the metropolitan area of Bologna is rapidly changing. Quality improvement initiatives are needed to reduce the burden for the universalistic Italian health care system generated by the rapidly-growing high-risk immigrant population.

## Introduction

Migration is an ancient phenomenon which is progressively changing the characteristics of the receiving populations. On a worldwide scale, the total number of migrants in the world exceeds 280 million, generated by two sets of factors, i.e.,“push factors” in native countries (food shortage, wars, civil wars, terrorism) and “pull factors” in host countries (economic booming, job opportunities, well-being) [1]. As of January 1 2022, over five million immigrants were regularly residing in Italy (8.4% of total population), but they account for over 12% in most industrialized areas.

Massive immigration is generating a series of critical issues to the universalistic Italian healthcare system, mainly in the area of chronic non-communicable diseases (NCD), particularly diabetes, due to the increasing number of cases at risk [2], the life-long duration and the cost of complications [3], as well as the cost of preventive and therapeutic measures to alleviate the burden of disease [4]. The relation between migration status and disease may be affected by genes, exposure in pre-migration life to poverty (epigenetic effects) [5], precarious health and sanitary conditions, eating habits, infections, rooted cultural customs retained in adulthood, exposures in post-migration life, as well as quality of health care and access to health services in countries of destination [6].

Also the clinical aspects and adherence to treatment may be different, as extensively investigated in different European contexts [6]. Agyemang et al. concluded that diabetes develops at a younger age in migrants, glycemic control remains poorer and the rate of micro and macro-vascular complications is higher than in the host European populations [7]. Italian data measuring the impact of migrants on diabetes, based on a cohort of over 8-million subjects confirmed that immigrants were on average 15 years younger and received a less intensive treatment, characterized by the use of cheaper antidiabetic drugs and less intensive monitoring of metabolic control and specialist visits [8]. This has long been associated with higher mortality, particularly in migrants coming from North and Sub-Saharan Africa [9], although mortality data are difficult to retrieve in selected cohorts, due to the “healthy migrant” effect, i.e., the selective process favoring migration in healthy individuals, and the “salmon bias”, i.e., the trend of sick migrants returning back to their home country before death [10], leading to underestimation [11]. Recent data did not confirm the higher mortality risks in “first generation” migrants with type 2 diabetes compared with Europeans [12], and differences were also found in relation to the country of origin.

The above considerations underline the importance of conducting studies aimed to investigate the relative contribution of immigrants to diabetes burden [13], considering the need for specific dietary and lifestyle interventions appropriate for their cultural environment [14], as well as culturally adapted lifestyle modification intervention trials.

In 2019, the Italian Ministry of Health launched a call intended to improve awareness about the importance of audit and feedback procedures to improve healthcare, and funded the metropolitan area of Bologna to merge data from the administrative and clinical databases that collect information regarding the epidemiology and treatment of diabetes and heart failure. It was an opportunity to investigate the impact of immigration on the clinical burden of diabetes on the Emilia-Romagna regional healthcare system, as well as the possible differences in surveillance and management, likely to results in future comorbidities.

## Materials and methods

### Patients

The current study includes prevalent patients diabetes registered inside the universalistic Italian Health System of the Local Health Authority of Bologna during the year 2019, identified by at least one of the following criteria: a) at least two prescriptions of glucose-lowering drugs (Code ATC A10xx); b) at least one hospitalization with the diagnosis code ICD-IX 250.xx (any position); c) exemption from co-payment because of diabetes (code 013); d) inclusion in the Diagnostic and Therapeutic Care Pathway (PDTA) in one of the two public endocrinological centers operating in the area of Bologna, according to a specific agreement of shared care. Patients must have survived up to December 31, 2019. This search provided data for 53,941 subjects on a total population of 884,507 inhabitants (crude prevalence, 6.1%).

All subjects were classified as born in/outside Italy, independent of their citizenship, according to the segment of the fiscal code related to place of birth. Specifically, patients with a segment matching a foreign country were classified as born outside Italy (immigrants), with the exception of those born in San Marino or in the former Italian colonies of Libya or Ethiopia (if born before 1946). Their general socio-demographic and clinical characteristics are reported in Table 1.

**Table 1:** Socio-demographic and clinical characteristics of the 2019 diabetes population, according to country of birth

	Total population (n = 53,949)	Italy-born (n = 47,235)	Foreign-born (n = 6706)	P Value
Males, n (%)	28,459 (52.7%)	25,440 (53.9%)	3019 (45.0%)	< 0.001*
Age (years)	71.4 [60.6–79.8]	73.0 [63.7–80.7]	54.8 [44.3–63.7]	< 0.001^§^
≤ 35, 36–50, 51–65, 66–80, > 80 (%)	3.2, 8.5, 24.5, 42.5, 21.3	2.2, 5.6, 22.2, 46.1, 24.0	10.6, 29.0, 40.7, 17.2, 2.5	< 0.001*
Body Mass Index (kg/m^2^)	29.5 ± 5.9 (n = 12,886)	29.6 ± 6.0 (n = 10,926)	28.7 ± 5.4 (n = 1960)	< 0.001^
Normal-weight, overweight, obesity (%)	19.2, 37.5, 43.3	18.6, 37.3, 44.0	22.3, 38.6, 36.1	< 0.001*
Diabetes visits, n (%)	21,518 (39.9%)	18,475 (39.1%)	3043 (45.3%)	< 0.001*
Cardiology visit, n (%)	14,378 (26.7%)	13,217 (28.0%))	1161 (17.3%)	< 0.001*
Electrocardiogram, n (%)	13,498 (25.0%)	12,400 (26.3%))	1098 (16.4%)	< 0.001*
Eye examination, n (%)	14,384 (26.7%)	12,948 (27.4%)	1436 (21.4%)	< 0.001*
Retinopathy screening, n (%)	4427 (8.2%)	3835 (8.1%)	592 (8.8%)	0.051*
HbA1c (mmol/mol)	48 [42–57]	48 [42–56]	50 [42–63]	< 0.001^§^
< 48, 48–52,53–63,64–74, ≥ 75 (%)	47.0, 17.2, 21.3, 8.4, 6.1	47.8, 17.5, 21.4, 8.0, 5.3	41.0, 14.8, 20.5, 11.5, 12.1	< 0.001*
Not measured, n (%)	10,192 (22.6%)	10,156 (21.5%)	2036 (30.4%)	< 0.001*
LDL-cholesterol (mg/dL)	105.9 ± 34.5	105.0 ± 34.1	114.4 ± 36.6	< 0.001^
< 70, 70–99, 100–129, ≥ 130 (%)	13.8, 32.6, 30.5, 23.1	14.1, 33.3, 30.3, 22.2	10.4,26.2,31.9,31.5	< 0.001*
Not measured, n (%)	15,753 (29.2%)	13,097 (27.7%)	2656 (39.6%)	< 0.001*
Albuminuria (median, mg/L)	8 [3–22]	8 [3–22]	7 [3–18]	< 0.001^§^
< 20, 21–200, ≥ 200 (%)	73.2, 21.7, 5.1	72.8, 22.1, 5,1	76.5, 18.1, 5,4	< 0.001*
Not measured, n (%)	23,845 (44.2%)	20,438 (43.3%)	3407 (50.8%)	< 0.001*
eGFR (mL/min × 1.73 m^2^)	73.0 ± 23.7	71.2 ± 23.1	90.2 ± 22.5	< 0.001^
CKD stage 1, 2, 3a, 3b, 4, 5 (%)	27.8, 43.5, 14.8, 9.3, 3.7, 0.9	24.0, 45.5, 15.7, 10.0, 3.9, 0.9	63.4, 25.5, 5.8, 3.1, 1.3, 0.9	< 0.001*
Not measured, n (%)	12,214 (22.6%)	9974 (21.1%)	2240 (33.4%)	< 0.001*

Data are expressed as mean ± standard deviation, as median [interquartile range] or as percentage (95% confidence interval)

Abbreviation: eGFR, estimated glomerular filtration rate

*Chi-square test

^§^Mann–Whitney test

^^^t-test

Data used for the study were retrieved from the databases of hospital admissions, specialty ambulatory visits, administered drugs, and laboratory tests performed by the Metropolitan laboratory Service that provides all analyses performed by public services in the area. Analyses performed by private laboratories, operating on a pay-per-service basis, were not available as were visits performed on a private practice basis.

In addition, we obtained data regarding the prevalence of diabetes and the relative average income of the resident population by city districts from municipal statistics.

### Data merging, anonymization and compliance with privacy rules

In order to merge different sources of information, while being compliant with privacy regulations, an anonymization tool was developed. The tool is a RESTful web service with anonymization and deanonymization endpoints. The anonymization algorithm ensures that for the same input patient ID, the same output anonymous ID is returned, and that for different input patient IDs, different output anonymous IDs are returned. For security compliance, the anonymization service accessible via secure authentication was performed by ICT personnel who did not analyse data for the study, and all the data are kept on a dedicated schema to which others cannot have access.

For all the data imported into the Easy-net project database, the anonymization procedure was applied as follows: the data was imported from the source, the anonymization service was called to replace the patient IDs with the anonymised patient IDs, and the original data was deleted to prevent users from tracing personal patient information.

### Statistical analysis

A descriptive analysis was initially performed on sociodemographic and clinical data by computing mean and standard deviation or median of continuous variables and frequency (%) of categorical variables. Comparison of socio-demographic and clinical characteristics of Italy-born and immigrant patients were carried out by chi-square test, t-test or Mann–Whitney test, according to the distributional features. The risk of diabetes in immigrant vs. Italy-born cohort was calculated by odds ratio (OR) and 95% confidence interval (95%CI), after adjustment for age and sex as confounding variables. All analyses were performed using Stata v.17.1.

### Ethical issues

The protocol was submitted to the local Ethic Committee of Bologna and approved after amendments (Protocol # 229.2018.Oss.AOUBo). Informed consent was waived by the Ethical Committee, considering the retrospective nature of the protocol, the number of cases under assessment and the complete anonymization of data.

## Results

### Socio-demographic and clinical data

The vast majority of the population was identified by antidiabetic drug prescription (80.5%); hospitalization codes for diabetes were retrieved in 8.1% of cases, exemption codes in 62.2%, PDTA participation in 26.3%. Foreign-born patients accounted for 12.4% of the total diabetes cohort (n = 6714, Table 1), a figure slightly higher than the prevalence of the immigrant population in the metropolitan area of Bologna (12.2%). Compared with the Italy-born population, they were identified less commonly by drug use (77.6% vs 81.0%) and hospitalization codes (6.9% vs. 8.2%), and more commonly by inclusion in shared care programs between diabetes centers and general practitioners (33.1 vs. 25.3), without differences in the rate of exemption codes. When related to the correspondent overall population living in the Bologna area, the prevalence of diabetes was identical (6.1%) in the two cohorts of foreign-born and Italy-born individuals. There was a moderate majority of males among the total and Italy-born diabetes populations (53.9%), whereas a prevalence of women was detected in the immigrant cohort (55.0%). The most striking difference was in age, with Italy-born subjects much older (by 18 years: 73.0 vs. 54.8) than individuals of the foreign-born cohort, and with a larger prevalence of obesity. More than half of patients did not comply with the standards of the agreed Diagnostic and Therapeutic Care Pathway (PDTA) for diabetes regarding attendance to diabetes units, cardiovascular (ECG) testing and retinopathy screening and/or assessment of metabolic control (Table 1). Median HbA1c indicated a good metabolic control in both groups; foreign-born patients showed higher values of LDL-cholesterol, similar values of albuminuria and a more preserved renal function. The rates of unmeasured biomarkers were always higher in foreign-born patients, ranging from 30.4% with unmeasured HbA1c to 50.8% of unmeasured albuminuria.

### Accrual of patients prevalent in 2019 and follow-up to 2021

**Fig. 1:**
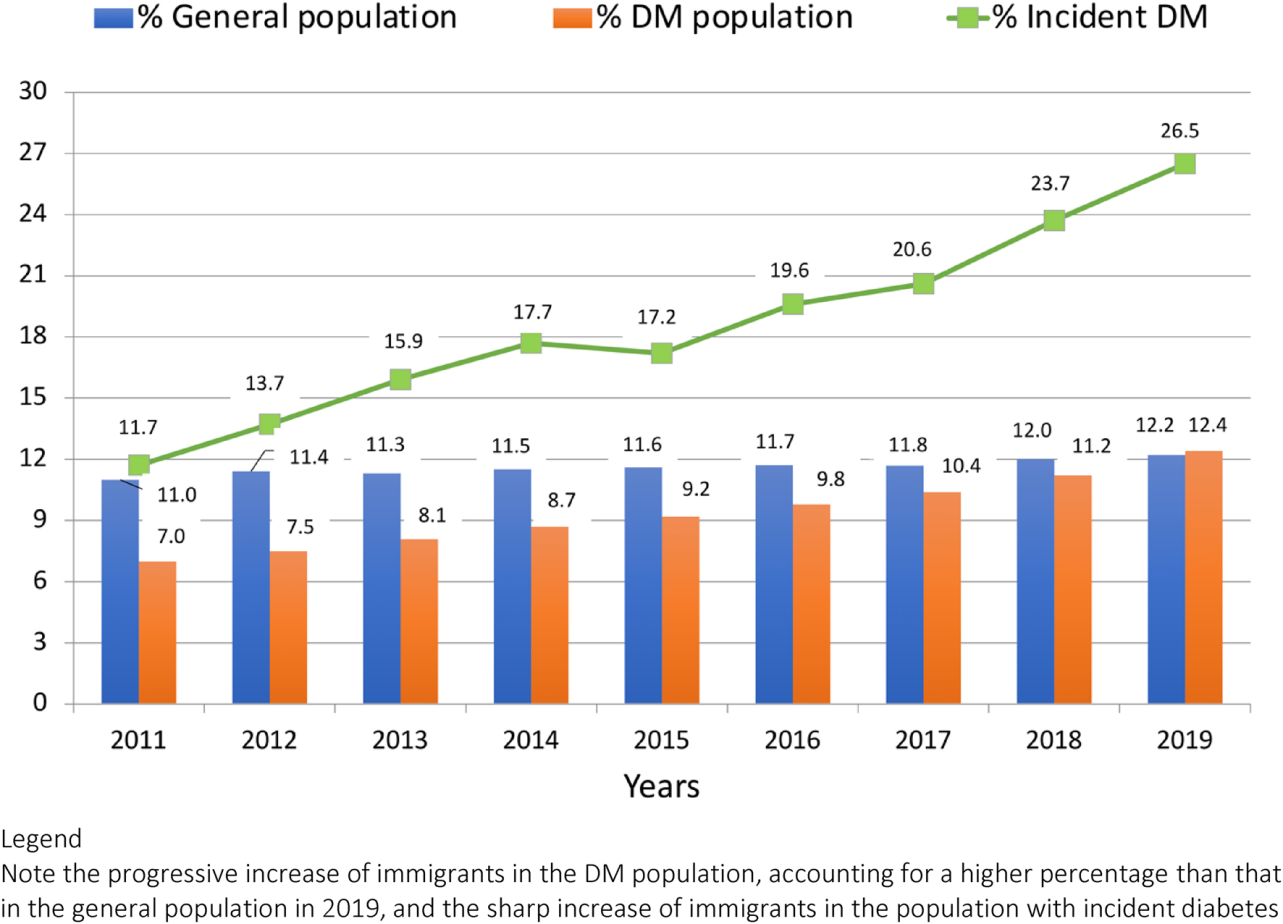
Percent of immigrants in the general population of the metropolitan area of Bologna and percent of prevalent and incident immigrants (line chart) in the population with diabetes included in the 2019 diabetes database (by year of accrual)

The year of accrual into the 2019 diabetes database was retrospectively investigated. Only 7% of foreign-born residents had diabetes in 2010, compared to 11.0% in the Italy-born cohort but from that year onwards immigrant cases with incident diabetes (median age, 49 vs. 65 in Italy-born individuals) increased systematically up to 26.5% in 2019 (Fig. 1), in both genders (from 8.9% to 21.0% in males and from 14.9% to 32.8% in females). When split by age group, the proportion of immigrants increased from 43.2% to 53.4% in the age group < 35 years, from 37.1% to 49.0% between 35 to 44 years, nonetheless displaying an increasing trend with time in all age groups (from 3.4% to 12.9% also in subjects aged 65–74).

A sensitivity analysis on the diabetes administrative databases of Bologna relative to 2011 and 2015 confirmed that the prevalence of immigrants on incident diabetes was much lower than in 2019 (11.8% and 16.1%, respectively, vs. 26.5% in 2019), increasing systematically by 1.8% per year from 2011 to 2019. Similarly, a prospective analysis in 2020 and 2021 confirmed the dramatic increase of foreign-born patients on incident diabetes, accounting for 30.2% and 27.5% of cases, respectively.

**Fig. 2:**
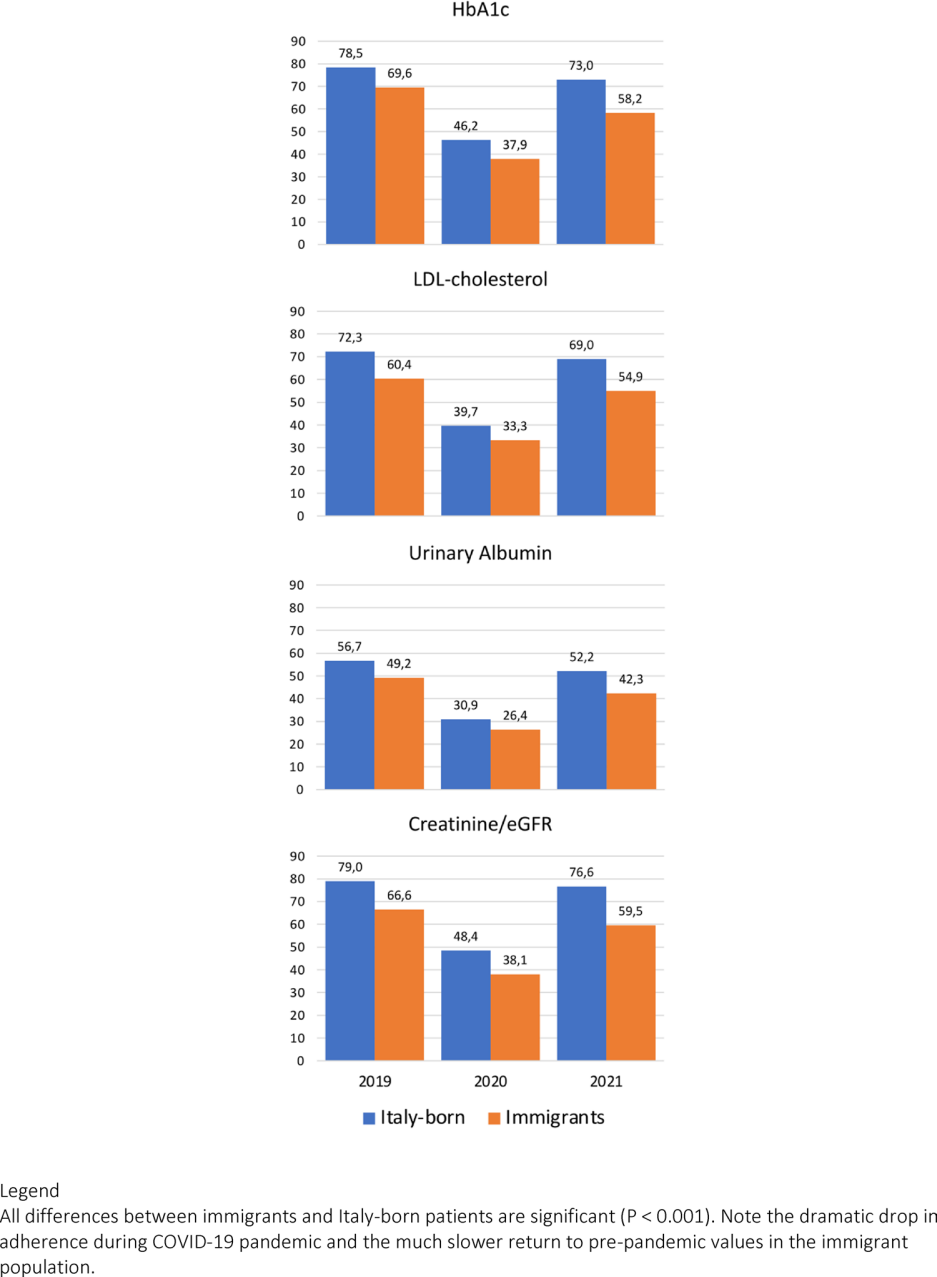
Percentage of cases with available data (one or more per year) reflecting adherence to Diagnostic and Therapeutic Care Pathway (PDTA) in subjects with diabetes residing in the metropolitan area of Bologna

The analysis of compliance with the agreed surveillance tests of the Diagnostic and Therapeutic Care Pathway (PDTA) showed a remarkable deterioration in 2020, as the effect of COVID-19 lock-down, and a partial return to 2019 compliance in 2021. In general, the effects of pandemia seem to have generated a larger impact in immigrant healthcare vs. Italy-born patients with diabetes (Fig. 2).

### Risk of diabetes according to country of origin and socio-demographic conditions

**Fig. 3:**
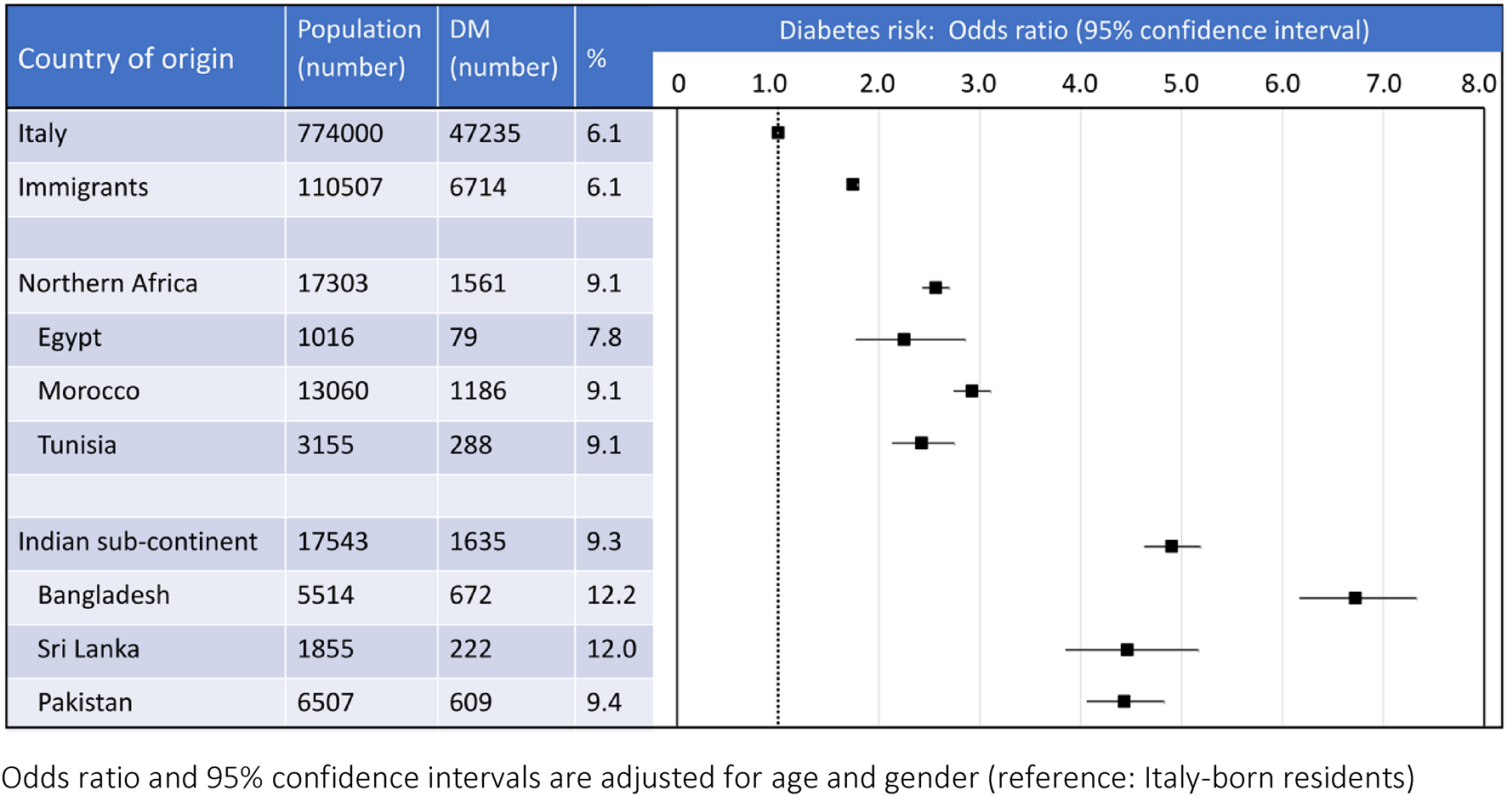
Diabetes prevalence and risk according to country of origin in subjects residing in the metropolitan area of Bologna

In the presence of a similar diabetes prevalence, the risk of diabetes was increased by 74% in immigrants, compared with Italy-born cohort (OR 1.74; 95% CI 1.69–1.79; Fig. 3), after adjustment for age and sex. The risk was particularly high in immigrants from the Mediterranean and sub-Saharan Africa (Northern Africa), (n = 1561; OR 2.56; 95% CI 2.43–2.52), comprising immigrants from Algeria, Egypt (OR 2.25; 95% CI 1.77–2.86), Libya, Morocco (OR 2.92; 95% CI 2.74–3.11), Western Sahara, South Sudan, Sudan, Tunisia (OR 2.42; 95% CI 2.13–2.75), as well as in immigrants from the Indian subcontinent (Southern-Central Asia; OR 4.90; 95% CI 4.63–5.19), comprising 17,543 patients with diabetes from Afghanistan, Bangladesh (OR 6.72; 95% CI 6.17–7.33), Bhutan, India, Kazakhstan, Kyrghyzstan, Maldive, Nepal, Pakistan (OR 4.43; 95% CI 4.06–4.83–3.11), Sri Lanka (OR 4.46; 95% CI 3.85–5.17), Tajikistan, Turkmenistan, Uzbekistan.

Notably, the risk of diabetes differed significantly inside the 18 districts of the Municipality of Bologna, being as low as 3.2% in more affluent areas and as high as 6.5% in more deprived, peripheral districts, mostly inhabited by immigrants (up to 25.8% of total residents). The prevalence of diabetes in these districts was inversely proportional to quintiles of income (r_s_ = − 0.933; P < 0.001), and directly proportional to the prevalence of immigrants (r_s_ = 0.915; P < 0.001).

### Treatment of diabetes and associated comorbidities

Only 6.7% of the total 2019 diabetes cohort was treated with the sole diet. This number was higher in immigrants (8.8%) than in Italy-born patients (6.4%; P < 0.05). In both cohorts (Table 2), metformin was used in approximately 60% of cases; small differences between cohorts, although significant, might stem from the use of metformin in association with other oral antidiabetic drugs. Sulfonylurea treatment declined in both cohorts (more rapidly in immigrants), the use of acarbose and pioglitazone was rare and stable. The use of DPP-4 inhibitors increased in both groups, but remained 3–4% lower in immigrants, whereas treatments with GLP-1 receptor agonists and SGLT-2 doubled. Unexpectedly, the use of SGLT-2 inhibitors was larger in immigrants, as was the use of basal insulin. The use of drugs of the ATC C group was much lower in immigrants, in keeping with their lower cardiovascular risk. This was consistent with the total number of cases (n = 3837; 7.1%) who had suffered an episode of hospital admission for heart failure in the period 2017–2019, corresponding to only 213 cases (3.2%) in the immigrant population vs. 3624 (7.7%) in the Italy-born cohort (P < 0.001). Notably, the Italy-born population with diabetes and heart failure was nearly 15 years younger than the corresponding immigrant population (mean ± SD, 79.1 ± 9.9 years vs. 65.0 ± 11.6; P < 0.001).

**Table 2:** Drug use in patients of the 2019 diabetes cohort in the metropolitan area of Bologna, according to immigrant status

Drug treatment	Alive by 31/12/2019			Alive by 31/12/2020			Alive by 31/12/2021		
	Italy-born (n = 47,235)	Immigrants (n = 6706)	P	Italy-born (n = 44,852)	Immigrants (n = 6647)	P	Italy-born (n = 42,573)	Immigrants (n = 6583)	P
Metformin, ATC A10BA, n (%)	27,239 (57.7%)	4117 (61.3%)	^^^	26,331 (58.7%%)	3839 (57.8%)	^^^	25,956 (61.0%)	3766 (57.2%)	^^^
Sulfonylureas,* ATC A10BB-BC, n (%)	5835 (12.4%)	827 (12.3%)		5332 (11.9%)	711 (10.7%)	^^^	5183 (12.2%)	689 (10.5%)	^^^
Pioglitazone,* ATC A10BG, n (%)	652 (1.4%)	94 (1.4%)		670 (1.5%)	103 (1.5%)		727 (1.7%)	128 (1.9%)	
Acarbose,* ATC A10BF, n (%)	778 (1.6%)	53 (0.8%)	^^^	681 (1.5%)	44 (0.7%)	^^^	603 (1.4%)	38 (0.6%)	^^^
DPP-4 inhibitors,* ATC A10BH, n (%)	3309 (7.0%)	332 (4.9%)	^	3845 (8.6%)	354 (5.3%)	^^^	4211 (9.9%)	372 (5.7%)	^^^
GLP-1 receptor agonists,* ATC A10BJ, n (%)	1837 (3.9%)	193 (2.9%)	^^^	2289 (5.1%)	253 (3.8%)	^^^	3147 (7.4%)	395 (6.0%)	^^^
SGLT-2 inhibitors,* ATC A10BK, n (%)	921 (1.9%)	161 (2.4%)	^^^	1168 (2.6%)	225 (3.4%)	^^^	1743 (4.1%)	304 (4.6%)	^^^
Basal insulin,* ATC A10AE, n (%)	8039 (17.0%)	1432 (21.3%)	^^^	8228 (18.3%)	1274 (19.2%)		8234 (19.3%)	1290 (19.6%)	
Basal-bolus insulin, ATC A10AB-C-D, n (%)	6184 (13.1%)	1054 (15.7%)	^^^	6378 (14.2%)	938 (14.1%)		6365 (15.0%)	948 (14.4%)	
Anti-hypertensive drugs, ATC C02, n (%)	3290 (7.0%)	201 (3.0%)	^^^	73,179 (7.1%)	187 (2.8%)	^^^	3242 (7.6%)	208 (3.2%)	^^^
Diuretics, ATC C03, n (%)	9225 (19.5%)	579 (8.6%)	^^^	9419 (21.0%)	570 (8.6%)	^^^	9741 (22.9%)	564 (8.6%)	^^^
Beta-blockers, ATC C07, n (%)	17,754 (37.6%)	1265 (18.8%)	^^^	17,944 (40.0%)	1284 (19.3%)	^^^	18,065 (42.4%)	1329 (20.2%)	^^^
Calcium-channel blockers, ATC C08, n (%)	9853 (20.9%)	868 (12.9%)	^^^	9920 (22.1%)	849 (12.8%)	^^^	10,089 (23.7%)	883 (13.4%)	^^^
ACE inhibitors/AT-1 blockers, ATC C09, n (%)	26,582 (56.3%)	2411 (35.9%)	^^^	26,246 (58.5%)	2361 (35.5%)	^^^	26,084 (61.3%)	2335 (35.5%)	^^^
Statins, ATC C10, n (%)	24,050 (50.9%)	2620 (39.0%)	^^^	23,984 (53.5%)	2585 (38.9%)	^^^	24,616 (57.8%)	2746 (41.7%)	^^^
Anti-platelet drugs, ATC B01AC, n (%)	18,839 (39.9%)	1490 (22.2%)	^^^	18,572 (41.4%)	1420 (21.4%)	^^^	18,394 (43.2%)	1460 (22.2%)	^^^
Neprelysin inhibitors, ATC C09DX04, n (%)	113 (0.2%)	7 (0.1%)	^^^	157 (0.4%)	7 (0.1%)	^^^	219 (0.5%)	9 (0.1%)	^^^

*With/without metformin

^^^Significantly different from the corresponding value in the Italy-born cohort (χ^2^ test, P < 0.05)

## Discussion

Our analysis of diabetes epidemiology and care in the metropolitan area of Bologna underlines critical issues arising in relation to diabetes risk in immigrants. The pattern of diabetes presentation is dramatically changing, with over 25% of new cases belonging to patients born outside Italy (first immigration individuals), characterized by a much younger age (by nearly 20 years), a larger prevalence of women and lower than normal adherence to the agreed metabolic evaluations and control visits, as well as different pharmacologic treatment. All these conditions are very likely to increase the future burden of diabetes in the population and the costs for the universalistic Italian healthcare system. These effects had been previously documented in large Italian databases [8], but the longitudinal approach used in this analysis reveals an acceleration of the process that requires immediate intervention.

In order to identify immigrants in the general population, we took advantage of the fiscal code, a unique identification code for all people living in Italy, which includes country of birth. Country of birth is a crude method for identifying ethnicity, a concept implying same origins or social environment, definite culture and customs, and a common language or religious heritage [15, 16], which risks becoming a vague measure of lifestyle habits as time since migration goes by. Nonetheless, using nationality or citizenship may bring even more critical issues as immigrants may have nationality and citizenship of the host country, but yet belong to ethnic groups with different behavior, religion and culture [17, 18]. This point is particularly critical in Italy, where citizenship may only be obtained after prolonged residency also by “second generation” migrants. Differences in epidemiological surveys on migrants to Italy are likely to stem from these differences in ascertainment methods.

The most important result of this study is the demonstration that immigrants account for a much larger proportion of incident diabetes cases than expected on the basis of population prevalence. In 2019 more than one in four cases of incident diabetes in the metropolitan area of Bologna was born outside Italy, a figure more than doubling the prevalence of immigrants in the total population. This excess of diabetes is even more larger considering the age distribution of immigrants, a population characterized by a proportion of three children (0–14 years) for any adult aged 65 or older [1]. The younger age of immigrants is very likely to reflect also a shorter duration of diabetes, underestimating the impact of migration on the retrospective analysis of accrual of incident diabetes in the years 2011–2019, as supported by the sensitivity analysis.

The evidence of high risk in women and in young individuals points to gestational diabetes as a possible source of error. A post-hoc analysis of diabetes risk in women under the age of 50 retrieved incident diabetes in the year 2019 retrieved an almost equal number of cases in immigrants (n = 537) vs. Italy-born cases (n = 538), turning into an odds ratio of 4.50 (95% confidence interval, 3.74–5.41). This risk, although high, is unlikely to offset the general conclusion, also considering that gestational diabetes remains a risk for future type 2 diabetes.

Our data confirm that the risk of diabetes is remarkably different according to the country of origin. In the presence of an overall prevalence of diabetes not different in immigrants and Italy-born individuals (6.1%), the risk was over 70% increased when adjusted for age and sex, but increased over five folds in selected cohorts. Similar data were originally observed in the Filipino population living in Rome, and referred to the development of obesity, Westernized food intake, reduced physical activity and years of residency in Italy [19], definitely affecting the risk of type 2 diabetes. Similarly, a high prevalence of diabetes was reported in Chinese first-generation migrants aged 16–59 years settled in Prato, in association with several comorbidities, despite similar awareness of impending risk [20] but much lower body size perception [21]. A higher risk was also observed in the prevalence of type 1 diabetes in children of North-African migrants in the Emilia-Romagna region (including Bologna) [22], also reported in a multicenter Italian study [23], possibly increasing the difference in overall diabetes prevalence compared with the Italy-born population. Unfortunately, we were unable to dissect the risk of type 1 vs. type 2 diabetes in our database, considering the progressive increase of type 2 diabetes in adolescents in relation to obesity and the possible occurrence of type 1 diabetes across different age groups, as well as uncertain reporting and the lack of definite markers in the metropolitan database.

We could also confirm the different level of adherence to agreed protocols of diagnostic and therapeutic care between immigrants and Italy-born individuals with diabetes, despite higher inclusion rates. The higher number of patients failing to comply with planned visits and the values of biochemical parameters were associated of a loss of stringent metabolic control among immigrants, with the exclusion of kidney function data, that were more favorable in migrants. The results confirm data previously collected in a larger Italian setting [8], as well as data observed in a different area of Emilia-Romagna region [24], but also in other countries and supported by a systematic review [25, 26]. As presented in Fig. 2, the adherence to diagnostic procedures in migrants was reduced further during the COVID-19 pandemic, when most control visits were moved from hospital to web or phone contact, and reflects lower awareness of health risks [27], as well as socio-economic and educational differences, also related to gender differences [28], that prevent adequate treatment [25, 27]. In general, our analysis suggests that appropriate care of the immigrant population remains a challenge for general practitioners and specialist centers, and specific strategies should be developed as part of the agreed PDTA of shared care. The evidence that tailored educational intervention may indeed improve diabetes management in migrants is weak [29], but attempts should be made to reduce future risks.

Treatment was also different in relation to migrant status. Contrary to what was observed in a previous study [8], the use of antidiabetic drugs, including insulin, was not systematically reduced, whereas the use drugs for the treatment of comorbidities was lower. This might stem from the perception of reduced cardiovascular risk, given the younger age of patients, leading to unacceptably high levels of LDL-cholesterol levels. Notably, during COVID-19 pandemic, when the follow-up of patients with chronic conditions was moved from hospital to phone consultations (starting on March 11, 2019), the adherence to the protocol dropped further, particularly in the immigrant population.

In summary, our study shows that, despite efforts made to extend the universalistic healthcare system to all persons residing in Italy, differences still exist that put a large number of people at high risk of comorbidities and future events. Barriers generated according to the three factors of Andersen’s behavioral model of healthcare use [30]—i.e., needs (awareness of disease, with particular evidence for chronic conditions); enabling conditions (educational level, socio-economic conditions); and predisposing factors (language proficiency, Western attitudes on family care, male–female roles, family values, religion)—appear to be fully operative and dictate management, as supported by the correlation between diabetes prevalence and income. The massive increase of immigrants among cases with incident diabetes prompts extensive revisiting of our procedures and a move from opportunistic interventions to proactive strategies.

## Data Availability

The datasets generated during and/or analyzed during the current study are not publicly available due privacy and hospital regulations and but are available from the corresponding author on reasonable request.
